# The reemergence of Aedes aegypti in Arizona.

**DOI:** 10.3201/eid0302.970223

**Published:** 1997

**Authors:** D M Engelthaler, T M Fink, C E Levy, M J Leslie


### The Reemergence of *Aedes aegypti* in Arizona

**To the Editor:** *Aedes aegypti*, primarily an urban, tropical mosquito, is a competent vector of dengue and yellow fever viruses. In the early 1900s, *Ae. aegypti* was found in every country in the Western Hemisphere except Canada. In the United States, repeated attempts to eradicate it have failed, and the mosquito is now well established in the southern states, from Texas to South Carolina, and more recently in Maryland and New Jersey (1,2).

Although the arid landscape of southern Arizona is an unlikely habitat for *Ae. aegypti*, these mosquitoes were identified in the cities of Tucson between 1931 and 1946 (3,4) and Yuma in 1951 (5). Elsewhere in the western United States, *Ae. aegypti* has been conspicuously absent, except for periodic reports of populations in New Mexico during this same period (3,6).

Beginning in 1969, the Arizona Department of Health Services initiated an arbovirus surveillance program involving state and local officials in routine monthly mosquito sampling between May and October of each year. Until 1994, no *Ae. aegypti* specimens had been identified in Arizona through routine surveillance, which involved adult collection with $CO_2$ and New Jersey light traps and larval dipping surveys, or through other mosquito research (7,8).

In August 1994, a University of Arizona entomology professor reported finding several *Ae. aegypti* in his Tucson backyard. Followup surveys in September and October 1994 by state and county health officials identified a number of *Ae. aegypti* in that same neighborhood as well as in central Tucson. During September 1995, additional specimens were collected in Nogales and again in Tucson. The adult mosquitoes were collected with $CO_2$ traps in various locations in these two cities. In Tucson, trapping from four of five sites yielded 85 adult *Ae. aegypti* (8.5/trapnight). In Nogales, trapping from two of four sites yielded 122 adults (12.2/trapnight). Trapping done earlier in 1995 at these sites yielded no *Ae. aegypti* adults.

Between 1994 and 1995, *Ae. aegypti* were trapped exclusively after the monsoon season (late July to early September), when late summer precipitation allowed for sufficient breeding conditions in backyards. However, in late March 1996, the Arizona Department of Health Services responded to a report of "ankle-biting" mosquitoes in central Tucson. Subsequently, two adult *Ae. aegypti* were trapped in the complainant's home. Since then, adult *Ae. aegypti* have been found in several new areas in and around Tucson (0-10/trapnight). Adult specimens have also been found for the first time in the Arizona-Mexico border towns of Douglas (<1/trapnight) and Naco (<1/trapnight; an additional 17 adults were collected by aspiration).

It is not certain whether *Ae. aegypti* mosquitoes were newly introduced in southern Arizona, or if they have been present at low, undetectable levels until favorable weather conditions allowed the population to proliferate. However, trapping data from the last three decades suggest the former.

Oviposition trapping has been the method of choice for *Ae. aegypti* surveillance (9). In Arizona hay infusion-enhanced oviposition traps were used (10). Because of the climate, initial attempts to use oviposition traps for *Ae. aegypti* surveillance were unsuccessful: the containers did not maintain enough water long enough for the hay infusion to attract egg-laying. Future oviposition trapping attempts will use variations on this trap, such as more infusion medium and/or larger containers, and will focus on careful trap placement. These changes may yield a more appropriate *Ae. aegypti* trap for use in the arid deserts of Arizona.

The establishment of populations of *Ae. aegypti* in Arizona is of particular concern to the local health services because of the presence of more than 400 laboratory-confirmed cases of dengue fever in the bordering Mexican state of Sonora in 1996 (R. Navarro Coronado, pers. comm.). While no cases of endemic dengue have been reported in Arizona, two imported cases were identified in 1994. Records show that between 1941 and 1946 nine cases of dengue fever were reported in Arizona's residents, eight of which were from the Tucson and Nogales areas. No exposure or travel history for those cases is available. Imported cases of yellow fever were reported southeast of Tucson in the late nineteenth century. The mere presence of infected patients allowed for possible endemic disease transmission, because of the simultaneous presence of *Ae. aegypti* populations. If, or when, new cases of dengue are identified in Arizona residents, this same predicament will again exist. *Ae. aegypti* surveillance throughout southern Arizona will be expanded in the coming years, and surveillance will continue for new dengue cases, imported or otherwise.

**David M. Engelthaler, T. Michael Fink, Craig E. Levy, and Mira J. Leslie**
Arizona Department of Health Services, Phoenix, Arizona, USA

### References

1. Sweeney K, Cantwell M, Dorothy J. The collection of *Aedes aegypti* and *Ae. albopictus* from Baltimore, Maryland. J Am Mosq Control Assoc 1988;4:381-82.
2. Donnelly J. *Aedes aegypti* in New Jersey. J Am Mosq Control Assoc 1993;9:238.
3. Bequaert J. *Aedes aegypti*, the yellow fever mosquito, in Arizona. Bulletin of the Brooklyn Entomological Society 1946;41:157.
4. Murphy D. Collection records of some Arizona mosquitoes. Entomological News 1953;14:233-8.
5. Richards CS, Nielsen LT, Rees DM. Mosquito records from the Great Basin and the drainage of the lower Colorado River. Mosquito News 1956;16:10-6.
6. Ferguson F, McNeel TE. The mosquitoes of New Mexico. Mosquito News 1954;14:30-1.
7. Smith HH, Janssen RJ, Mail GA, Wood SA. Arbovirus activity in southern Arizona. Am J Trop Med Hyg 1969;18:448-54.
8. McDonald JL, Sluss TP, Lang JD, Roan CC. Mosquitoes of Arizona. Tucson (AZ): University of Arizona Agricultural Experiment Station; 1973 Technical Bulletin No.: 205.
9. Service MW. Mosquito ecology: field sampling methods. New York (NY): John Wiley & Sons, 1976.
10. Rieter P, Amador M, Colon N. Enhancement of the CDC ovitrap with hay infusions for daily Monitoring of *Aedes aegypti* populations. J Am Mosq Control Assoc 1991;7:52-5.Treatment of Exudative Pharyngitis

**To the Editor**: The dispatch by Izurieta et al. (Emerg Infect Dis 1997;1:65-8) reporting exudative pharyngitis possibly due to *Corynebacterium pseudodiphtheriticum* was very interesting, especially with the resurgence of diphtheria in the former Soviet Union. However, I was somewhat surprised at the treatment received by the 4-year-old patient whose case is reported. Erythromycin is an effective antibiotic in diphtheria, but it is secondary in importance to diphtheria antitoxin.

The presence of a thick grayish white adherent pseudomembrane, adenopathy and cervical swelling, and low grade fever should certainly provoke a high index of suspicion of diphtheria, especially in a child who has not received pediatric immunization. The diagnosis of diphtheria is primarily made presumptively on clinical grounds and confirmed by the recovery of toxigenic *Corynebacterium diphtheriae* by the laboratory.

Antitoxin treatment cannot wait for laboratory confirmation. Prompt administration of antitoxin is important because diphtheria toxin binds rapidly and irreversibly to tissue sites. Delay in initiating antitoxin treatment is associated with increased incidence of myocarditis, paralysis, and death. Also, it would have been good practice to have placed this child in isolation until the diagnosis was established by the laboratory. The primary care physician in this case is indeed fortunate that the patient did not have diphtheria; the results could have been tragic.

**Paul D. Ellner, Ph.D.**
Professor Emeritus of Microbiology and Pathology, Columbia University College of Physicians and Surgeons

**Reply to P.D. Ellner:** We agree that diphtheria antitoxin should be administered promptly on the basis of a presumptive clinical diagnosis of respiratory diphtheria. Because laboratory confirma-tion may be delayed, the decision to treat with antitoxin and the dose of antitoxin must be based on the site and size of the diphtheritic membrane, the degree of toxicity, and the duration of illness (1,2).

Respiratory diphtheria is rare in the United States. From 1980 to 1995, only 41 cases were reported (zero to five cases in any given year) (3). With this low incidence, the likelihood that a patient with membranous pharyngitis has respiratory diphtheria is low. In addition, membranous pharyngitis could be associated with infections by other organisms such as streptococci, Epstein Barr virus, *Candida albicans*, *Borrelia vincenti*, *Herpes simplex* virus, *Arcanobacterium hemoliticum*, nontoxigenic *Corynebacterium diphtheriae*, and *Corynebacterium pseudodiphtheriticum* as in the case we reported (4-10).

The diagnosis and clinical management of exudative pharyngitis with a pseudomembrane in a country where diphtheria is extremely rare

